# Utilizing Alcohol Septal Ablation for Mitigating Left Ventricular Outflow Tract Obstruction in Cardiac Amyloidosis: A Case Report

**DOI:** 10.7759/cureus.62633

**Published:** 2024-06-18

**Authors:** Kyrillos Girgis, Ari Feinberg, Danielle Retcho, Tony Elias, Allen George, Grettel Gonzalez Garcia, Rafail Beshai, Gouthami Chennu, Reenal Patel, Marc Cohen

**Affiliations:** 1 Internal Medicine, Newark Beth Israel Medical Center, Newark, USA; 2 Cardiovascular Disease, Newark Beth Israel Medical Center, Newark, USA; 3 Internal Medicine, Rowan University School of Osteopathic Medicine, Camden, USA; 4 Cardiovascular Disease, Virtua, Camden, USA; 5 Internal Medicine, Jefferson Stratford Hospital, Stratford, USA; 6 Cardiology, Newark Beth Israel Medical Center, Newark, USA

**Keywords:** treatment modality, unique presentation, left ventricular outflow obstruction (lvot), cardiac amyloidosis, alcohol septal ablation (asa)

## Abstract

Alcohol septal ablation (ASA) has been widely used in relieving the left ventricular outflow tract (LVOT) obstruction caused by hypertrophic obstructive cardiomyopathy (HOCM). There is limited data about the utility of ASA in cases of cardiac amyloidosis with LVOT obstruction. Our patient is 71-year-old male with a history of multiple myeloma complicated by cardiac amyloidosis and end-stage renal disease on hemodialysis who presented from the dialysis center due to hypotension. The patient was admitted to our hospital for further workup. He underwent echocardiography that showed severely elevated LVOT gradient pressures and the decision was made to proceed with ASA, which led to significant improvement in the LVOT gradient pressures and the patient being able to tolerate his dialysis sessions.

## Introduction

Cardiac amyloidosis is considered the most common type of restrictive cardiomyopathy [1]. It results from the extracellular accumulation of a noxious component known as amyloid, which is a combination of improperly folded proteins and other matrix-forming components such as proteoglycans and glycosaminoglycans [2]. Cardiac amyloidosis is caused by the accumulation of either amyloid transthyretin (ATTR), which is the most common cause, or amyloid light (AL) chain proteins that are produced by abnormal plasma cells as in the case of multiple myeloma [2]. Cardiac amyloidosis can be discovered incidentally while evaluating for different organ involvements in systemic amyloidosis or it can present with symptoms of exertional dyspnea, palpitations, or chest pain [3]. Patients may experience classic heart failure symptoms, such as dyspnea, orthopnea, paroxysmal nocturnal dyspnea, and lower limb edema [3].

Herein, we present a unique case of cardiac amyloidosis presenting with left ventricular outflow tract (LVOT) obstruction, where the use of alcohol septal ablation led to significant improvement in the patient’s hemodynamics and hence his symptoms.

## Case presentation

Our patient is a 71-year-old male with multiple myeloma complicated by cardiac amyloidosis, ventricular tachycardia status post-automatic implantable cardioverter-defibrillator (AICD) placement, systemic hypertension, and end-stage renal disease on hemodialysis. He presented from the dialysis center due to hypotension for which the dialysis session was terminated. The patient stated that he had frequent episodes of dizziness and endorsed similar episodes of hypotension during dialysis sessions. The patient denied chest pain or shortness of breath. Upon arrival, he was alert, oriented, and in mild distress.

Vital signs were significant for low blood pressure of 90/58 mmHg. Physical exam was within normal limits, including cardiac exam, which showed normal heart rate, regular rhythm, equal peripheral pulsations, and absence of distended jugular veins or peripheral edema. An electrocardiogram (EKG) showed an atrial-paced rhythm with a normal heart rate. Labs showed normal hemoglobin, brain natriuretic peptide (BNP) of 3,000 pg/mL (normal: < 100 pg/mL), no significant electrolyte abnormalities, and normal thyroid-stimulating hormone (TSH).

**Figure 1:**
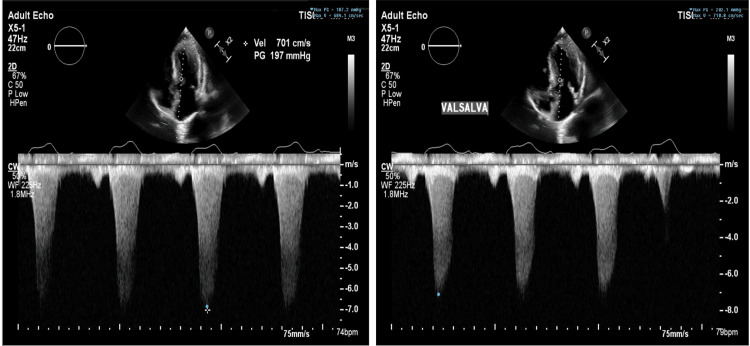
Initial left ventricular outflow tract (LVOT) gradient before alcohol septal ablation at rest and with Valsalva.

The patient was admitted to the hospital for further workup. Transthoracic echocardiography (TTE) (Video 1) and transesophageal echocardiography (TEE) (Videos 2, 3) showed severe concentric left ventricular hypertrophy with interventricular septal thickness of 3 cm associated with systolic anterior motion of the anterior leaflet of the mitral valve. The LVOT peak gradient was 187 mmHg at rest and 200 mmHg with Valsalva (Figure 1). These findings are consistent with hypertrophic obstructive cardiomyopathy (HOCM)-like physiology with a severe gradient across the LVOT due to severe proximal septal thickening. 

**Video 1:** Transthoracic echocardiogram (TTE) long parasternal view pre-alcohol septal ablation.

**Video 2:** Trans-esophageal echocardiogram (TEE) three-chamber view pre-alcohol septal ablation.

**Video 3:** Trans-esophageal (TEE) three-chamber view with color Doppler pre-alcohol septal ablation.

**Figure 2:**
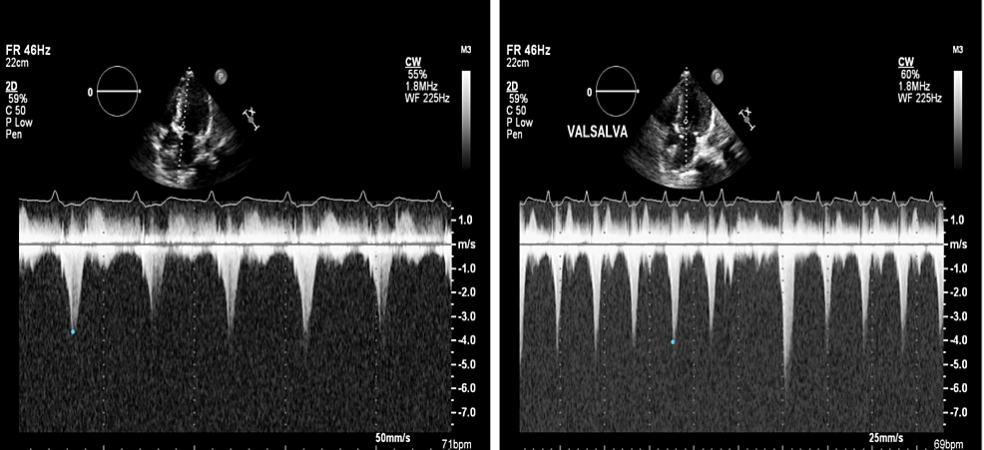
Improvement in the left ventricular outflow tract (LVOT) gradient post-alcohol septal ablation at rest and with Valsalva.

Given the symptomatic LVOT obstruction and the fact that our patient was not a candidate for myomectomy because of his co-morbidities and old age, the decision was made to proceed with alcohol septal ablation (ASA) (Videos 4-9). The patient is status post ASA of the first septal perforator. Before the start of the procedure, there was a gradient of 100 mmHg across the LVOT. Post ASA, the LVOT gradient disappeared. TTE two days post ASA showed basal anteroseptal wall hypokinesis with improvement in the degree of systolic anterior motion of the mitral valve. The peak LVOT gradient at rest was 52 mmHg and with Valsalva was 66 mmHg (Figure 2). 

**Video 4:** Angiogram showing the right coronary artery (RCA).

**Video 5:** Angiogram showing the left anterior descending (LAD) and first septal perforator.

**Video 6:** Angiogram showing balloon placement in the first septal perforator.

**Video 7:** Angiogram showing distal alcohol injection in the first septal perforator.

**Video 8:** Angiogram showing proximal alcohol injection in the first septal perforator.

**Video 9:** Angiogram showing the first septal perforator status post-alcohol ablation.

The patient reported improvement of his dizziness symptoms and tolerated hemodialysis sessions better than before. No complications were found in the immediate or early post-operative follow-up.

## Discussion

Cardiac amyloidosis is an uncommon disease. The incidence varies depending on the etiology. AL amyloidosis occurs in around 10% of multiple myeloma patients, with cardiac involvement in 50%-70% of those. The yearly incidence of AL amyloidosis is one in 100,000 [4]. Amyloid deposition has the potential to cause a variety of cardiac problems. Direct interstitial infiltration induces increased ventricular wall thickness that leads to diastolic dysfunction [2]. In AL amyloidosis, amyloid can build up in arterioles, causing angina or even myocardial infarctions. Light chains can potentially cause direct damage to cardiac cells by releasing reactive oxygen species [3].

ASA has become an increasingly common minimally invasive therapeutic option for HOCM since it was first described in 1994 [5]. In order to remove the dynamic outflow obstruction, this catheter-based technique uses pure alcohol injected into the septal perforator, causing a controlled infarction of the hypertrophied septum, which causes a notable clinical improvement in the patient's symptoms [5].

Initially, ASA causes an immediate decrease in outflow gradient, primarily due to stunning rather than structural changes. However, this reduction may be temporary, with the gradient recurring after one to three days [6]. Over three to 12 months, a more significant and permanent reduction in gradient occurs due to scar formation and left ventricular remodeling [5,6]. Cardiac imaging techniques reveal decreased myocardial mass and wall thickness post ASA, along with improvements in systolic and diastolic function parameters [7].

Our case is considered unique as our patient has significant LVOT obstruction secondary to cardiac amyloidosis and not due to HOCM. There is limited data about the use of ASA in cases of LVOT obstruction secondary to cardiac amyloidosis. Despite the challenging nature of our case, our team opted for ASA as a therapeutic intervention. Remarkably, following the procedure, the patient exhibited a marked and sustained improvement in both symptoms and hemodynamics. The targeted infusion of alcohol into septal coronary artery branches induced localized myocardial necrosis and fibrosis, effectively reducing LVOT obstruction, alleviating our patient’s symptoms of dizziness, and allowing him to better tolerate the dialysis sessions.

This successful outcome underscores the potential efficacy of ASA in managing LVOT obstruction secondary to cardiac amyloidosis, offering hope for similar patients facing limited treatment options. This case highlights the importance of individualized care and innovative approaches in managing complex cardiovascular conditions such as cardiac amyloidosis, ultimately leading to improved patient outcomes and quality of life.

## Conclusions

ASA is very effective in improving the hemodynamics, echocardiographic features, and hence symptoms caused by LVOT obstruction even in cases without hypertrophic obstructive cardiomyopathy as in our patient who has LVOT obstruction secondary to cardiac amyloidosis. Larger studies are required to corroborate our findings.
